# Effects of antioxidant co‐supplementation therapy on spermatogenesis dysfunction in relation to the basal oxidation–reduction potential levels in spermatozoa: A pilot study

**DOI:** 10.1002/rmb2.12450

**Published:** 2022-02-27

**Authors:** Kazumitsu Yamasaki, Masahiro Uchida, Noriko Watanabe, Tatsuji Ihana, Yukari Ishiguro, Shinnosuke Kuroda, Teppei Takeshima, Yasushi Yumura, Makiko Mieno, Kaoru Yoshida, Teruaki Iwamoto, Hiroyuki Nishiyama

**Affiliations:** ^1^ Department of Urology Tsukuba Gakuen Hospital Tsukuba Japan; ^2^ Male Infertility Center for Human Reproduction Sanno Hospital Minato‐ku Japan; ^3^ Oak Clinic Ginza Chuo‐ku Japan; ^4^ Center for Human Reproduction and Gynecologic Endoscopy Sanno Hospital Minato‐ku Japan; ^5^ Department of Urology, Reproduction Center Yokohama City University Medical Center Yokohama Japan; ^6^ Department of Medical Informatics Center for Information Jichi Medical University Shimotsuke Japan; ^7^ Faculty of Biomedical Engineering Toin University of Yokohama Yokohama Japan; ^8^ Department of Urology Faculty of Medicine University of Tsukuba Tsukuba Japan

**Keywords:** 8‐hydroxy‐2′‐deoxyguanosine, antioxidant, infertility, male, oxidation–reduction, sperm count

## Abstract

**Purpose:**

In this pilot study, the authors compared the effects of antioxidant co‐supplementation therapy and methylcobalamin therapy in patients with impaired semen quality.

**Methods:**

Eighty‐four subjects who visited male infertility clinics and showed abnormal semen test results were randomly subjected to one of the two therapies: antioxidant co‐supplementation therapy with vitamin C, vitamin E, coenzyme Q10, and flaxseed oil or methylcobalamin therapy. The oxidation–reduction potential (ORP) and 8‐hydroxy‐2′‐deoxyguanosine levels were used as indicators of oxidative stress levels in semen. Semen analysis was also performed.

**Results:**

The authors obtained results from 67 patients who had completed 3 months of treatment. Neither antioxidant co‐supplementation therapy nor methylcobalamin therapy changed the semen parameters significantly (except for the sperm concentration, which was increased by the latter therapy). When the pre‐treatment ORP value in semen was higher than the cutoff value, both therapies significantly increased the sperm concentration. The 8‐hydroxy‐2′‐deoxyguanosine level did not yield any meaningful predictive value with regard to increased sperm concentrations.

**Conclusions:**

Both antioxidant co‐supplementation therapy and methylcobalamin therapy increased the sperm concentration in patients with impaired semen quality when the basal ORP levels in their semen were elevated.

## 1 INTRODUCTION

As living organisms use oxygen, every biological reaction involves a redox state, including spermatogenesis and sperm function. Oxidative stress (OS) inside and outside the sperm is thought to cause male infertility by causing DNA damage and peroxidation of cell membrane lipids.^1^ One approach for resisting this damage is to inhibit excessive oxidation by scavenging reactive oxygen species or suppressing their generation through the intake of dietary or supplemental ingredients.

A Cochrane meta‐analysis performed in 2019 showed that antioxidant supplements used to treat male infertility increased live‐birth and pregnancy rates; however, they did not affect miscarriage rates and led to moderate adversities associated with the gastrointestinal system.^2^ Some studies reported a significant positive effect of antioxidant therapy on semen parameters, outcomes of assisted reproductive therapy, and live‐birth rate as well as results of advanced sperm function test including OS measurements.^3^, ^4^ In contrast, another meta‐analysis of results published over the past decade, which was conducted to determine whether there were sufficient data to justify administration of antioxidants to infertile men, found that only 30 clinical studies examined the effects of small‐molecule antioxidant administration in infertile men.^5^ Of these, only 43.3% were case–control studies, 66.7% enrolled more than 40 patients, and 40% administered a single antioxidant. Therefore, additional studies are needed to clearly establish and define the utility of antioxidant‐based therapies for male infertility.

An example of an antioxidant supplement is the oxidized form of ubiquinone (CoQ10). Ubiquinone is isolated from the inner mitochondrial membrane and prokaryotic cell membranes and is widely known to be involved in electron transfer within the membrane. Organisms that use ubiquinone as a respiratory chain electron carrier (e.g., humans) can synthesize ubiquinone. No studies have demonstrated its efficacy as a drug, but the use of ubiquinone as a dietary supplement has rapidly gained attention. A previous placebo‐controlled, double‐blind randomized trial in patients with idiopathic asthenozoospermia showed that exogenous administration of coenzyme Q10 (CoQ10) increased the levels of both CoQ10 and ubiquinol to further improve sperm motility.^6^

Vitamin E prevents the chain reaction of lipid oxidation by forming vitamin E radicals by dissipating free radicals. The generated vitamin E radicals are regenerated into vitamin E by antioxidants, such as ascorbic acid (vitamin C). Ascorbate can end these chain radical reactions through electron transfer reactions; the oxidized form of ascorbate is relatively unreactive and does not cause cell damage. Vitamin E and C are widely used as antioxidants in food additives. Administration of them, in conjunction with glutathione, significantly decreased 8‐hydroxy‐2′‐deoxyguanosine (8‐OHdG) levels in spermatozoa and increased the sperm count.^7^ Recent studies have examined the combinations of some of these antioxidant supplements and demonstrated their effectiveness in treatment of men with oligoashtenozoospermia.^8^

Methylcobalamin is considered a coenzyme for the methionine synthase enzyme and plays a role in methylating important functional macromolecules and molecules such as DNA, RNA, neurotransmitters, lipids, and proteins. Methylcobalamin has been shown to positively affect semen quality by increasing the functionality of reproductive organs and decreasing homocysteine toxicity.^9^ Recent studies suggested that methylcobalamin has weak antioxidant properties, but there is insufficient evidence of its relationship with oxidative stress in humans.^10^, ^11^

A readily available, inexpensive supplement may be beneficial for treating infertile male patients with few effective treatment options. However, only a few small‐scale studies have been conducted in this field. This study was conducted to evaluate patients who visited male infertility clinics and showed abnormal semen test results. We compared the effects of a combination of supplements with an established strong antioxidant capacity to those of an active control, methylcobalamin, which has long been used to treat male infertility, on these patients.

## 2 MATERIALS AND METHODS

### 2.1 Study design

This pilot study was conducted at the Center for Human Reproduction and Gynecologic Endoscopy, Sanno Hospital, International University of Health and Welfare, Tokyo, Japan, in collaboration with the Department of Urology, Tsukuba Gakuen Hospital, Tsukuba, Japan, and Reproduction Center, Yokohama City University Medical Center, Yokohama, Japan, between April 2018 and March 2020. The study was designed in a prospective one‐to‐one concealed to randomization manner using the envelope method in a controlled, non‐blinded, and open‐label fashion.

### 2.2 Study subjects

A total of 84 patients who visited male infertility clinics were included in this study. Adult males who visited the above three centers between May 2018 and February 2019 were screened based on the following criteria before enrollment in the study—those with either oligospermia, asthenozoospermia, or oligoasthenozoospermia (i.e., at least one of the following semen characteristics according to WHO 2010 reference value^12^: sperm concentration <15 million cells/mL, total sperm motility <40%) were considered to be eligible for the study; those with normal semen parameters and an immediate history of antioxidant supplementation (<3 months) or with severe oligozoospermia (sperm concentration <1 million cells/mL) and severe hypospermia (semen volume <1 mL) were deemed as ineligible. Severe oligozoospermia and hypospermia posed technical challenges in the measurement of oxidation–reduction potential (ORP) and 8‐OHdG. The semen diagnosis was made by at least two semen analysis, which were repeated up to four times when the variability was large. Patients who refused randomization were excluded from the study.

### 2.3 Study protocol

Physicians specializing in male infertility obtained a full medical history from each study participant and performed a physical examination (general and local genitals) of each participant. It was confirmed that none of the subjects had systemic diseases such as diabetes and/or autoimmune diseases that could cause elevated serum OS, or cryptorchidism and reproductive tract infections. Also, none of them had a treatment history of radiation therapy or chemotherapy. Varicocele was diagnosed based on clinical examination and confirmed by color Doppler analysis. Clinical varicocele severity was graded according to the criteria described by Dubin and Amelar.^13^ According to the study protocol, there were 28 subjects in each group, and 84 subjects were included; however, because of a shortage of eligible candidates within the planning period, the final number of subjects was 80. Participants were allocated at a balanced one‐to‐one ratio to either the antioxidant co‐supplementation therapy group with 80 mg/day vitamin C, 40 mg/day vitamin E, 150 mg/day coenzyme Q10, and 220 mg/day flaxseed oil (two tablets of SO support II; Partners, Yokohama, Japan) or methylcobalamin therapy group with 1500 µg/day methylcobalamin (three tablets of Mecobalamin; Sawai Pharmaceutical, Osaka, Japan).^8^, ^14^ The participants underwent their assigned therapy for three months with monthly visits to outpatient clinics for compliance checks.

### 2.4 Serum hormone level measurement

Serum luteinizing hormone (LH), follicle‐stimulating hormone (FSH), and total testosterone (T) levels were measured using a chemiluminescent immunoassay (SRL, Inc.). The reference ranges for LH, FSH, and T were 0.79–5.72 mIU/ml, 2.00–8.30 mIU/ml, and 1.31–8.71 ng/ml, respectively. The intra‐assay coefficients of variation for LH, FSH, and T were 3.03%, 3.74%, and 5.13%, respectively; inter‐assay coefficients were 1.84%, 0.43%, and 3.99%, respectively.

### 2.5 Semen analysis

After complete liquefaction, each sample was evaluated to determine the ejaculate volume. An aliquot of the sample was examined to determine the sperm concentration, total sperm count, total sperm motility, and total motile sperm count (TMC) using the World Health Organization Fifth Edition guidelines (WHO, 2010).^12^ Semen analyses were conducted using the Sperm Motility Analysis System (SMAS™; DITECT Ltd.).

### 2.6 Oxidative stress assessment

OS was assessed by measuring both the static oxidation–reduction potential (ORP) of liquefied semen samples and 8‐OHdG levels in the seminal plasma. ORP, also known as the redox balance, was measured using the MiOXSYS^TM^ System (Aytu Bioscience, Inc.). Briefly, a 30‐µL aliquot of liquefied semen was loaded onto a test sensor that had been pre‐inserted into the MiOXSYS analyzer. To control for differences in the sperm count, ORP values were normalized by dividing the ORP by the sperm concentration (10^6^/ml) and are represented as mV/10^6^ sperm/ml.^15^ A cutoff value of 2.59/mV/10^6^/mL was used to differentiate oligozoospermia from normal, asthenozoospermia, and teratozoospermia conditions.^16^ According to this classification, patients were grouped into high‐ORP (>2.59 mV/10^6^ sperm/ml) and low‐ORP (≤2.59 mV/10^6^ sperm/ml) groups.

The 8‐OHdG levels, which are widely considered as key biomarkers of oxidative DNA damage, were measured using an enzyme‐linked immunosorbent assay kit (Japan Institute for the Control of Aging), according to the manufacturer's instructions. Briefly, 50 µl of seminal plasma was mixed with 50 µL of 8‐OHdG monoclonal antibody in a microtiter plate pre‐coated with 8‐OHdG. After overnight incubation, the plates were washed with phosphate‐buffered saline, horseradish peroxidase‐conjugated anti‐mouse IgG antibody was added, and the plates were further incubated. The color reaction product was detected by spectrophotometry at 450 nm, and the concentration of 8‐OHdG was calculated from a standard curve.

### 2.7 Statistical analysis

Statistical analysis was performed using JMP statistical software v.14.20 (SAS Institute). As our study variables were not normally distributed (Shapiro–Wilk test, *p* < 0.05), continuous variables were presented as medians and interquartile ranges (25th and 75th percentile) and analyzed using the Wilcoxon rank sum test or Wilcoxon signed‐rank test. Categorical variables between groups were compared using chi‐square test or Fisher's exact test when the tables were too sparse. The best cutoff value, sensitivity, specificity, and area under the curve were calculated using receiver operating characteristic (ROC) analysis. Statistical significance was set at *p* < 0.05.

## 3 RESULTS

The study subjects reported no adverse effects caused by either of the regimens during or after six months of the trial. Of the 80 participants, three patients were excluded later because of withdrawal of informed consent or if they did not meet the inclusion criteria for semen analysis (normozoospermia). Table 1 shows a comparison of the data for physical assessments, serum hormone levels, semen parameters, and levels of two types of OS markers (ORP and 8‐OHdG) between the antioxidant co‐supplementation therapy and methylcobalamin‐treated groups. The respective measurements did not significantly differ between groups. We also compared the semen parameters and OS markers between subjects with and without varicocele; again, there was no significant difference (Table S1).

**TABLE 1:** Baseline patient characteristics, serum hormone levels, semen parameters, ORP, and levels of 8‐OHdG

Characteristic	Antioxidants	Methylcobalamin	*p*
	(*n* = 40)	(*n* = 37)	
Age (y)	36.5 (32–41.8)	36 (32.5–42)	0.610*
Testicular volume^†^ (ml)			
Right	16 (14–20)	18 (14–21.75)	0.697*
Left	17 (14–20)	18 (14–21)	0.652*
Variables of varicocele			
Varicocele laterality (n)			0.825**
None	29	28	
Unilateral, right	3	3	
Unilateral, left	5	5	
Bilateral	3	1	
Varicocele grade (*n*)			
Right			0.754**
None	34	33	
Grade 1	0	0	
Grade 2	4	2	
Grade 3	2	2	
Left			0.376**
None	32	31	
Grade 1	2	3	
Grade 2	6	2	
Grade 3	0	1	
Past varicocelectomy (n)			
Yes	10	11	0.498**
No	30	26	
Semen parameters			
Semen volume^†^ (ml)	2.75 (1.74–3.4)	2.45 (1.55–3.3)	0.361*
Sperm concentration^†^ (n × 10^6^/ml)	16.8 (5.8–49.3)	15.4 (8.5–31.2)	0.729*
Sperm motility^†^ (%)	21.4 (9.6–34.1)	25.7 (14.7–33.9)	0.534*
TMC^†^ (n × 10^6^)	10.3 (3.2–25.6)	9.1 (2.8–28.8)	0.737*
ORP^†^ (mV/10^6^ sperm/ml)	1.51 (0.49–5.44)	1.30 (0.36–2.74)	0.600*
8‐OHdG^†^ (μmol/dl)	10.8 (9.7–12.1)	10.8 (8.5–14.4)	0.815*
Serum hormones			
Testosterone^†^ (ng/ml)	4.15 (3.10–5.56)	4.35 (3.05–5.86)	0.705*
LH^†^ (IU/L)	4.2 (2.58–5.3)	3.6 (3.1–5.6)	0.820*
FSH^†^ (IU/L)	4.46 (3.5–7.55)	5.42 (4.1–8.3)	0.506*

Abbreviations: 8‐OHdG, 8‐hydroxy‐2’‐deoxyguanosine; FSH, follicle‐stimulating hormone; LH, luteinizing hormone; ORP, oxidation–reduction potential; TMC, total motile sperm count.

^†^Median values (25th–75th percentile).

* Wilcoxon rank sum test.

** Chi‐squared corrected test.

### 3.1 Effect of antioxidant supplementation

Sixty‐seven participants completed the assigned three months of drug therapy. Table 2 summarizes the semen parameters, ORP, and 8‐OHdG levels of participants before and after treatment with antioxidant co‐supplementation or methylcobalamin. Antioxidant co‐supplementation significantly reduced sperm motility, albeit with a slight difference. Whereas the sperm concentration increased significantly after methylcobalamin therapy, its increase was insignificant after antioxidant co‐supplementation. Neither antioxidant co‐supplementation nor methylcobalamine yielded significant changes for either type of OS marker.

**TABLE 2:** Changes in semen parameters and ORP/10^6^ sperm in both treatment groups. Statistical assessments were performed not only on changes within the treatment group (Wilcoxon signed‐rank sum test), but also on post‐treatment results between the two groups (Wilcoxon rank sum test)

Parameters		Antioxidants			Methylcobalamin		
		(*n* = 36)			(*n* = 31^†^)		
	Baseline	3 months	*p*§	Baseline	3 months	*p*§	*p*
Semen volume^‡^ (ml)	2.75 (1.7–3.4)	2.55 (1.6–3.95)	0.776	2.45 (1.6–3.3)	2 (1.3–4.2)	0.297	0.955
Sperm concentration^‡^ (n × 10^6^/ml)	16.8(6.0–49.3)	19.1 (8.6–33.8)	0.927	14.0 (5.3–27.6)	19.0 (8.8–31.0)	0.017	0.945
Sperm motility^‡^ (%)	21.4 (8.7–32.9)	20.8(11.2–40.6)	0.036	24.5 (10–32.5)	15.5 (7.2–34.0)	0.261	0.297
TMC^‡^ (n × 10^6^)	10.3 (3.2–25.9)	9.8 (2.8–23.5)	0.747	8.9 (2.1–14.0)	6.8 (3.1–15.9)	0.462	0.421
ORP^‡^ (mV/10^6^ sperm/ml)	1.51 (0.40–5.07)	2.04 (0.85–3.33)	0.866	1.95 (0.64–3.70)	3.26 (1.43–6.02)	0.254	0.121
8‐OHdG^‡^ (μmol/dl)	10.7 (8.9–120.6)	10.6 (9.1–13.0)	0.314	10.6 (8.7–14.2)	10.3 (8.9–13.0)	0.856	0.567

Abbreviations: 8‐OHdG, 8‐hydroxy‐2’‐deoxyguanosine; ORP, oxidation–reduction potential; TMC, total motile sperm count.

†The number of samples used for assessing sperm motility, total motile sperm count, and ORP was not 31, but only 30, 30, and 29, respectively.

^‡^Median values (25th–75th percentile).

^§^ Wilcoxon signed‐rank sum test.

^¶^ Wilcoxon rank sum test.

### 3.2 Pre‐treatment ORP measurement and effect of antioxidants

Pre‐treatment semen parameters were examined after dividing the participants into two groups, those with low and high ORP, according to the cutoff value of the pre‐treatment ORP measurement (Table 3). The semen volume was significantly larger in the high‐ORP group than in the low‐ORP group. In contrast, the sperm concentration and TMC were significantly higher in the low‐ORP group than in the high‐ORP group. There was no apparent difference in 8‐OHdG levels between the two groups.

**TABLE 3:** Comparison of pre‐treatment semen parameters and 8‐OHdG levels between the low‐ and high‐ORP groups

Characteristic	Low ORP	High ORP	*p* ^†^
	(*n* = 52)	(*n* = 22)	
Semen volume^‡^ (ml)	2.13 (1.51–3.18)	2.95 (2.5–3.86)	0.0249
Sperm concentration^‡^ (n × 10^6^/ml)	28.0 (12.3–51.3)	5.7 (4.4–11.7)	<0.0001
Sperm motility^‡^ (%)	24.3 (12.4–32.5)	23.6 (7.0–38.0)	0.8453
TMC‡ (*n* × 10^6^)	13.4 (4.1–34.5)	4.9 (1.2–9.2)	0.0019
8‐OHdG^‡^ (μmol/dl)	10.9 (9.7–13.4)	10.2 (8.5–11.7)	0.1853

Abbreviations: 8‐OHdG, 8‐hydroxy‐2’‐deoxyguanosine; ORP, oxidation–reduction potential; TMC, total motile sperm count.

^‡^Median values (25th–75th percentile).

^†^ Wilcoxon rank sum test.

We then examined whether antioxidant co‐supplementation and/or methylcobalamin had different effects on semen parameters depending on the pre‐treatment ORP levels. As shown in Tables 4 and 5, antioxidant co‐supplementation yielded significant increases in both the sperm concentration and TMC only in the high‐ORP group. The low‐ORP group showed no change in any semen parameters. However, methylcobalamin treatment significantly increased the sperm concentration in the high‐ORP group.

**TABLE 4:** Changes in semen parameters via treatment in the low‐ORP group. Statistical assessments were performed not only on changes within the group (Wilcoxon signed‐rank sum test), but also on post‐treatment results between the two treatment groups (Wilcoxon rank sum test)

Parameters	Antioxidants (*n* = 24)			Methylcobalamin (*n* = 20)			
	Baseline	3 months	*p* ^†^	Baseline	3 months	*p* ^†^	*p* ‡
Semen volume^§^ (ml)	2.35 (1.56–3.33)	2.1 (1.1–3.83)	0.945	1.9 (1.43–3.03)	2 (1.23–4.5)	0.131	0.571
Sperm concentration^§^ (n × 10^6^/ml)	38.6 (15.6–52.6)	21.5 (7.5–44.4)	0.287	16.6(9.3–28.3)	19 (9.3–27.6)	0.216	0.612
Sperm motility^§^ (%)	23.8 (11.3–32.9)	21.3 (15.9–40.6)	0.098	23.3 (9.7–31.5)	11.4 (7.5–32.3)	0.870	0.081
TMC§ (n × 10^6^)	21.3 (4.6–45.7)	12.6 (4.7–28.7)	0.419	9.0 (2.3–17.5)	5.6 (2.9–13.2)	0.674	0.207

Abbreviations: ORP, oxidation–reduction potential; TMC, total motile sperm count.

^†^Wilcoxon signed‐rank sum test.

^§^Median values (25th–75th percentile).

^‡^ Wilcoxon rank sum test.

**TABLE 5:** Changes in semen parameters via treatment in the high‐ORP group. Statistical assessments were performed not only on changes within the group (Wilcoxon signed‐rank sum test), but also on post‐treatment results between the two treatment groups (Wilcoxon rank sum test)

Parameters	Antioxidants (*n* = 12)			Methylcobalamin (*n* = 9^†^)			
	Baseline	3 months	*p* ^‡^	Baseline	3 months	*p* ^‡^	*p* ^§^
Semen volume^¶^ (ml)	2.95 (2.55–4.36)	3.15 (2.15–3.95)	0.664	2.7 (1.9–3.62)	2.1 (1.8–3.25)	0.215	0.176
Sperm concentration^¶^ (n × 10^6^/ml)	6.1 (4.3–11.3)	12.7 (8.9–26.3)	0.043	5.2 (4.90–12.23)	15.4 (10.3–41.3)	0.008	0.303
Sperm motility^¶^ (%)	12.0 (6.1–39.0)	15.7 (4.4–38.7)	0.204	28.7 (12.7–41.4)	16.1 (3.6–48.7)	0.195	0.939
TMC¶ (n × 10^6^)	4.6 (1.1–5.7)	7.6 (2.2–16.5)	0.027	5.6 (2.3–9.9)	7.4 (1.9–29.8)	0.641	1.000

Abbreviations: ORP, oxidation–reduction potential; TMC, total motile sperm count.

^†^The number of samples used for the analysis of sperm motility and TMC was not 9 as indicated in the table, but 8 instead.

^‡^Wilcoxon signed‐rank sum test.

^¶^Median values (25th–75th percentile).

^§^ Wilcoxon rank sum test.

**FIGURE 1:**
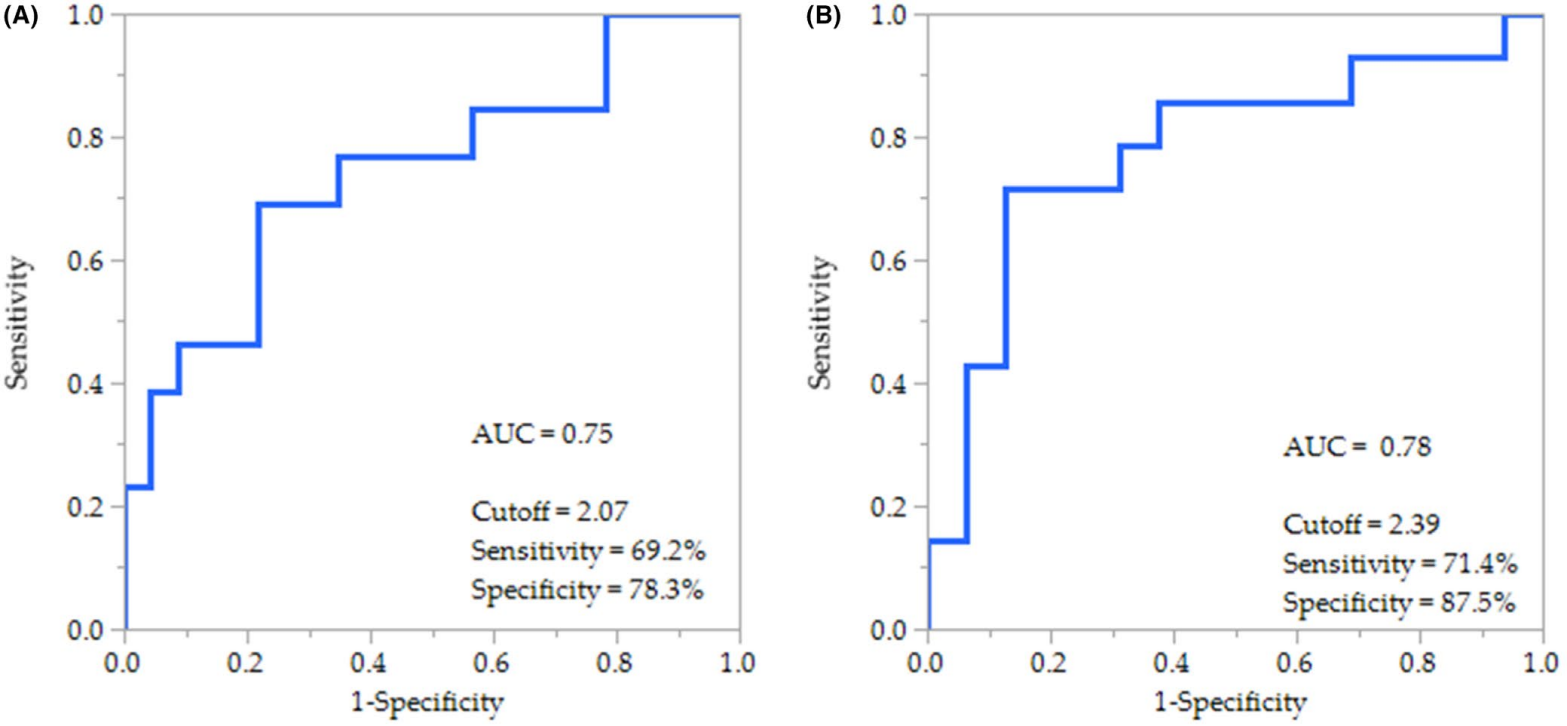
Area under the curve (AUC)‐receiver operating characteristic (ROC) of the pre‐treatment ORP group in predicting the improvement in sperm concentration by (A) the antioxidant co‐supplementation or (B) methylcobalamin treatments

Based on the significant increase in the semen concentration only in the high‐ORP group, we next performed ROC analysis of the pre‐treatment ORP in predicting improvement in the sperm concentration (Figure 1). The definition of improvement was that the post‐treatment sperm concentration was at least 150% higher than the pre‐treatment sperm concentration. The ROC curve yielded a cutoff value of 2.07 mV/10^6^ sperm/ml with 69.2% sensitivity and 78.3% specificity (area under the curve [AUC] =0.75) for antioxidant co‐supplementation. For methylcobalamin treatment, the ORP achieved 71.4% sensitivity and 87.5% specificity at a cutoff value of 2.39 mV/10^6^ sperm/ml (AUC = 0.78). We also conducted ROC analysis of the pre‐treatment 8‐OHdG level, another OS marker. 8‐OHdG did not yield meaningful predictive values for the improvement in sperm concentration, showing a cutoff value, sensitivity, specificity, and AUC of 11.80 μmol/dl, 92.3%, 39.1%, and 0.60, respectively, for the antioxidant co‐supplementation treatment, and 14.83μmol/dl, 35.7%, 94.1,% and 0.50, respectively, for methylcobalamin treatment (data not shown). There was no apparent correlation between ORP and 8‐OHdG in the pre‐ and post‐treatment samples (Figure S1).

When the ORP values before and after treatment were compared in cases where the sperm concentration was increased after 3 months of treatment and cases where no such increase was observed, there was no change in the former cases in either the antioxidant co‐supplementation or methylcobalamin treatment groups (Table 6). In the latter cases, the ORP values were significantly increased after 3 months of treatment.

**TABLE 6:** Changes in ORP (mV/10^6^ sperm/ml) via treatment in sperm concentration‐improved and not‐improved groups

Improvement of concentration	Antioxidants				Methylcobalamin			
	Baseline	3 months	P†	n	Baseline	3 months	*p* ^†^	*n*
Yes	2.64 (1.22–12.91)^‡^	2.69 (0.72–4.95)^‡^	0.109	13	2.74 (1.62–8.10)^‡^	4.39 (1.77–6.15)^‡^	0.542	13
No	1.20 (0.33–1.97)^‡^	1.53 (0.81–3.29)^‡^	0.049	23	1.03 (0.24–2.07)^‡^	2.43 (1.37–6.14)^‡^	0.029	16

Abbreviations: ORP, oxidation–reduction potential.

^†^Wilcoxon signed‐rank sum test.

^‡^Median values (25th–75th percentile).

Additionally, we investigated whether the two treatments had different effects on semen parameters and OS markers depending on the condition of the participants, such as whether they had varicocele. As shown in Table [Link], [Link] and [Link], [Link], antioxidant co‐supplementation did not significantly change the semen parameters or OS markers regardless of the varicocele condition. In contrast, methylcobalamin therapy increased the sperm concentration only in participants without varicocele (Table S2).

## 4 DISCUSSION

We compared the effects of antioxidant co‐supplementation therapy on semen parameters with methylcobalamin therapy in subjects who visited male infertility clinics and showed impaired semen quality. The antioxidant co‐supplementation contained CoQ10, vitamin C, and vitamin E, which have direct antioxidant actions, along with flaxseed oil, which is abundant in polyunsaturated fatty acids and exhibits indirect antioxidant activity. In contrast, methylcobalamin is considered to exert positive effects on semen quality and sperm physiology in multiple ways, including by exerting antioxidant activity.^9^ In addition to assessing the semen parameters before and after drug administration, we also measured ORP and 8‐OHdG to assess the OS status in sperm and examined whether these measurements can be used to identify patients in whom these antioxidant supplements may be beneficial.

As shown in Table 2, although methylcobalamin administration significantly increased the sperm concentration, antioxidant co‐supplementation did not change any semen parameters, except for sperm motility, which showed a slight decrease. Methylcobalamin may have a positive effect on sperm concentration by modulating the methylation state of the macromolecules/molecules in sperm. In contrast, antioxidant co‐supplementation appeared to affect neither the semen parameters nor the two OS markers, ORP and 8‐OHdG. The effect of antioxidants on male fertility remains controversial. Although several studies have reported favorable effects of antioxidant treatment on male infertility, others have shown that antioxidant treatment either has no influence or a negative influence on male infertility.^17^, ^18^ Our findings, however, provide evidence that antioxidant treatment has no impact on male infertility. This conflicting situation may be either attributed to the extensive heterogeneity associated with such studies or to the “antioxidant paradox” in which administration of large doses of dietary antioxidants to human subjects has little or no preventive or therapeutic effect. Nevertheless, OS has been implicated in several human diseases, including male infertility.^19^, ^20^ This may be because endogenous antioxidant defenses in the human body are complex, interlocked, and carefully regulated, and the body's “total antioxidant capacity” appears to be unresponsive to high doses of dietary antioxidants. Alternatively, in many studies, antioxidant supplementation was ineffective because the subjects with a baseline oxidative damage status were already well‐nourished with optimal levels of these antioxidants.

Based on the possibility that the response to antioxidant supplementation may differ depending on the baseline OS condition of the subjects, we divided the patients into two groups according to their pre‐treatment ORP levels to determine whether antioxidant administration had different effects on semen parameters in these two groups. To determine the effects of the treatments, pre‐treatment semen parameters and 8‐OHdG values were compared between the low‐ and high‐ORP groups. As shown in Table 3, the semen volume was significantly lower, and sperm concentration and TMC were significantly higher in the low‐ORP group than in the high‐ORP group. The ORP cutoff value of 2.59/mV/10^6^/ml has been shown to differentiate oligozoospermic samples from others in subjects with both infertile and fertile patients.^16^ This result indicates that even in infertile patients, the ORP value of semen may be negatively correlated with the sperm concentration, resulting in the same correlation with TMC, although the relationship between ORP and semen volume showed the opposite trend for unknown reasons.

Tables 4 and 5 show the effects of antioxidant co‐supplementation and methylcobalamin on semen parameters in the low‐ and high‐ORP groups. Antioxidant co‐supplementation significantly increased the sperm concentration and TMC in the high‐ORP group but not in the low‐ORP group, suggesting that high‐ORP levels indicate high levels of baseline OS in the sperm, which can be ameliorated by antioxidant supplementation. Interestingly, methylcobalamin also increased the sperm concentration (but not TMC) only in the high‐ORP group. It has been shown that methylcobalamin has positive effects on semen quality through multiple mechanisms,^9^ but the reason for this increase in sperm concentration only in the high‐ORP group is unclear. One possibility is that methylcobalamin exerts such effects through its recently reported antioxidant property; however, there is insufficient evidence to confirm that methylcobalamin acts as an antioxidant in the human body.^10^ Moreover, the serum level of methylcobalamin after administration at 1500 µg/day is likely insufficient to exert its antioxidant properties. According to the drug interview form provided by the manufacturer, the maximum serum concentration of methylcobalamin after administration of the dose used in this study was 0.62 nM, which is much lower than 5 µM, the lowest concentration by which methylcobalamin asserted the antioxidant property in an *in vitro* assay system.^11^ The relationship between methylcobalamin and ORP should be further examined.

ROC analysis revealed the clinical value of pre‐treatment ORP levels in predicting improvements in the sperm concentration with these treatments (Figure 1). Using these ORP cutoff values, it may be possible to select patients with male infertility who will benefit from antioxidant supplementation while avoiding its overuse, leading to reductive stress.^21^ Interestingly, even antioxidant co‐supplementation and methylcobalamin treatments did not decrease the ORP level in the semen sample of subjects whose sperm concentration was improved (Table 6). This observation appears to contradict the aforementioned hypothesis that high‐ORP levels represent an elevation in the OS status and that antioxidant supplementation is effective for ameliorating this elevation. The reason for this contradiction is not clear; however, the elevated OS status in semen may reflect a deficiency of specific intrinsic antioxidants, such as vitamin C, vitamin E, and CoQ10, and the antioxidant co‐supplementation and methylcobalamin treatments overcame the deficiency, whereas the semen OS status overall remained stable because of the complex and fine endogenous antioxidant balance.^19^

Interestingly, differences were observed between the two OS markers, ORP and 8‐OHdG. Subjects were divided into two groups according to their pre‐treatment ORP levels, but pre‐treatment 8‐OhdG levels did not differ between the groups (Table 3). In addition, we did not observe any apparent correlations between ORP and 8‐OhdG in both the pre‐treatment and post‐treatment samples (Figure S1). Thus, these two markers do not appear to represent the same aspect of sperm OS status. ORP, also known as the redox balance, is a direct measure of OS, as it describes the relative proportion of ROS to reductants (antioxidants).^15^ ORP levels are significantly negatively correlated with the sperm concentration, sperm motility, normal morphology, and TMC.^21^ In contrast, formation of 8‐OHdG is widely considered as a key biomarker of oxidative damage^22^, ^23^ and some studies showed that 8‐OHdG levels in the sperm are associated with semen parameters in patients with male infertility.^24^, ^25^ As expected, these two markers did not show consistent results, as it has been suggested that a single assay cannot be used to diagnose OS considering the multi‐dimensional nature of OS.^26^ As inferred from ROC analysis, however, ORP may be superior to 8‐OHdG in terms of selecting better markers that indicate suitable subjects for antioxidant supplementation (Figure 1). Notably, ultrafiltration of samples followed by the addition recovery test using a standard 8‐OHdG substance is recommended for certifying the accuracy of the measurement according to the manufacturer's manual when the 8‐OHdG enzyme‐linked immunosorbent assay kit is applied for blood serum and blood plasma samples.^27^ We did not perform these procedures to measure 8‐OHdG in seminal plasma samples; therefore, the result of our measurement may be less accurate, although in a study using the same measurement procedures, seminal plasma 8‐OHdG was increased in patients with varicocele, which was reduced by varicocelectomy.^24^

It has been reported that varicocele is associated with impaired semen quality and increased OS in sperms, both of which can be ameliorated by varicocelectomy.^24^, ^28^, ^29^ In this study, semen samples from participants with varicocele showed neither decreased semen parameters nor elevated basal levels of ORP and 8‐OHdG (Table S1). The reason for these observations may be explained by selection bias as follows: participants diagnosed without varicocele included those who had received varicocelectomy prior to inclusion in this study to improve semen quality, and it is possible that participants with varicocele had a tendency to avoid surgery because their semen parameters were relatively mildly impaired, indicating that the elevation of OS in their semen was relatively mild. This may also explain why antioxidant co‐supplementation did not change the semen parameters and OS markers in semen samples from participants with varicocele (Tables S2 and S3). Methylcobalamin may have increased the sperm concentration irrespective of varicocele, although the increase was not significant in participants with varicocele because of the small number of patients.

In conclusion, although this was a small and short time‐frame clinical interventional study which limits the ability to conduct subgroup analyses, our data indicate that both antioxidant co‐supplementation therapy with vitamin C, vitamin E, CoQ10, and flaxseed oil as well as methylcobalamin therapy can improve the sperm concentration in patients with impaired semen quality when the basal ORP levels in their semen are elevated. Therefore, ORP may be more useful for assessing the need for therapeutic antioxidant supplementation in patients with male infertility compared to using the OS marker 8‐OHdG.

## CONFLICT OF INTEREST

Kazumitsu Yamasaki, Masahiro Uchida, Noriko Watanabe, Tatsuji Ihana, Yukari Ishiguro, Shinnosuke Kuroda, Teppei Takeshima, Yasushi Yumura, Makiko Mieno, Kaoru Yoshida, Teruaki Iwamoto, and Hiroyuki Nishiyama declare that they have no conflict of interest. The antioxidant co‐supplement (SO Support II) and methylcobalamin were provided by Partners. This company did not play any role in the design of the study, collection, analysis, interpretation of data, writing of the manuscript, or in the decision to publish the results.

## HUMAN RIGHTS STATEMENT AND INFORMED CONSENT

All procedures followed were in accordance with the ethical standards of the responsible committee on human experimentation (institutional and national) and with the Helsinki Declaration of 1964 and its later amendments. Written informed consent was obtained from all the patients for inclusion in the study.

## ANIMAL RIGHTS

This article does not contain any studies with animal subjects performed by any of the authors.

## APPROVAL BY THE ETHICS COMMITTEE

This study was approved by the Institutional Review Board of the International University of Health and Welfare (No. 17‐S‐24, March 22, 2018), Yokohama City University (No. B180600058, July 9, 2018), and Tsukuba Gakuen Hospital (No.18–01, April 18, 2018).

## CLINICAL TRIAL REGISTRY

This was not a clinical trial.

## Supporting information

Fig S1

Table S1

Table S2

Table S3
