# Supplementary material for: Effects of antioxidant co‐supplementation therapy on spermatogenesis dysfunction in relation to the basal oxidation–reduction potential levels in spermatozoa: A pilot study
Source: Reprod Med Biol. 2022 Feb 27;21(1):e12450. doi: 10.1002/rmb2.12450 (PMC8967282; doi:10.1002/rmb2.12450)
Supplement: Supplementary file 1 — Fig S1 [file RMB2-21-e12450-s003.docx]

| 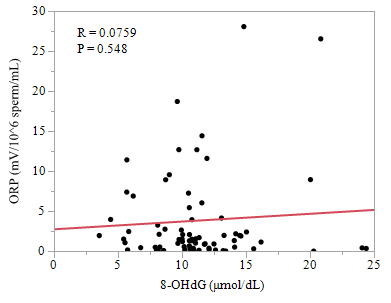 | 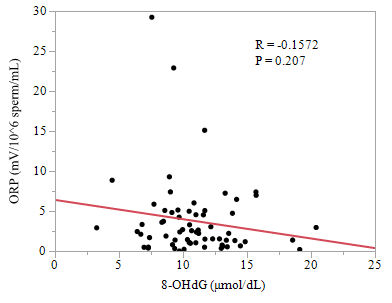 |
| --- | --- |
| (**a**) | (**b**) |

**Figure S1.** Correlation between 8-OHdG and ORP in (a) pre-treatment or (b) post-treatment samples. Correlation coefficients and P values from linear regression analysis are shown.
