# Supplementary material for: Effects of antioxidant co‐supplementation therapy on spermatogenesis dysfunction in relation to the basal oxidation–reduction potential levels in spermatozoa: A pilot study
Source: Reprod Med Biol. 2022 Feb 27;21(1):e12450. doi: 10.1002/rmb2.12450 (PMC8967282; doi:10.1002/rmb2.12450)
Supplement: Supplementary file 2 — Table S1 [file RMB2-21-e12450-s002.docx]

**Table S1.** Comparison of baseline semen parameters and OS markers between subjects with or without varicocele. Participants with left or bilateralvaricoceles were grouped into the varicocele group regardless of varicocele grade.

| **Characteristic** | **No-Varicocele** | **Varicocele** | **P*** |
| --- | --- | --- | --- |
|  | **(n = 57)** | **(n = 20)** |  |
| Semen parameters |  |  |  |
| Semen volume† (mL) | 2.5 (1.6-3.35) | 2.9 (1.94-4.01) | 0.137 |
| Sperm concentration† (n x10^6^/mL) | 14.4 (6.1-34.9) | 16.7 (6.3-42.1) | 0.714 |
| Sperm motility† (%) | 23.5 (9.5-32.9) | 28.6 (18.4-36.9) | 0.113 |
| TMC† (n x 10^6^) | 8.9 (1.7-24.2) | 11.9 (4.6-38.8) | 0.142 |
| ORP† (mV/10^6^ sperm/mL) | 1.39 (0.36-3.96) | 1.59 (0.47-3.47) | 0.985 |
| 8-OHdG† (μmol/dL) | 10.8 (8.7-12.3) | 11.0 (10.0-13.4) | 0.605 |

Abbreviations: ORP, oxidation-reduction potential; 8-OHdG , 8-hydroxy-2’-deoxyguanosine; TMC, total motile sperm count.

†Median values (25th–75th percentile)

*Wilcoxon rank-sum test
