# Supplementary material for: Effects of antioxidant co‐supplementation therapy on spermatogenesis dysfunction in relation to the basal oxidation–reduction potential levels in spermatozoa: A pilot study
Source: Reprod Med Biol. 2022 Feb 27;21(1):e12450. doi: 10.1002/rmb2.12450 (PMC8967282; doi:10.1002/rmb2.12450)
Supplement: Supplementary file 3 — Table S2 [file RMB2-21-e12450-s001.docx]

**Table S2.** Changes in semen parameters via treatment in subjects without varicocele. Participants with left or bilateralvaricoceles were grouped into the varicocele group regardless of varicocele grade. Statistical assessments were performed not only on changes within the group (Wilcoxon signed-rank sum test), but also on post-treatment results between the two treatment groups (Wilcoxon rank-sum test).

| **Parameters** | **Antioxidants (n = 27)** | | |  |  | **Methylcobalamin (n = 24)** | | |  |  |
| --- | --- | --- | --- | --- | --- | --- | --- | --- | --- | --- |
|  | **Baseline** | **3 months** | **P**† |  |  | **Baseline** | **3 months** | **P**† |  | **P**‡ |
| Semen volume§ (mL) | 2.7 (1.7-3.4) | 2.2 (1.5-3.5) | 0.534 |  |  | 2.1 (1.53-3.25) | 2 (1.3-3.3) | 0.522 |  | 0.850 |
| Sperm concentration§ (nx10^6^/mL) | 17.5 (5.8-50) | 21 (10-34) | 0.963 |  |  | 12.2 (6.0-25.9) | 18.0 (9.6-30.3) | 0.011 |  | 0.978 |
| Sperm motility§ (%) | 16 (8.5-31.7) | 20.7 (10-40.2) | 0.059 |  |  | 24.3 (7.4-33.6) | 11.5 (7.3-36.9) | 0.478 |  | 0.586 |
| TMC§ (n x10^6^) | 5.9 (1.2-25.9) | 9.1 (2.2-21.9) | 1.000 |  |  | 8.8 (1.2-10.5) | 7.2 (1.4-13.9) | 0.197 |  | 0.586 |
| ORP§ (mV/10^6^ sperm/mL) | 1.51 (0.33-5.44) | 2.39 (0.81-4.83) | 0.657 |  |  | 1.70 (0.89-9.25) | 2.78 (1.47-5.69) | 0.205 |  | 0.370 |
| 8-OHdG§ (μmol/dL) | 10.8 (9.6-12.1) | 10.7 (9.2-13.1) | 0.496 |  |  | 10.1 (8.2-12.0) | 9.6 (8.4-13.4) | 0.923 |  | 0.144 |

Abbreviations: 8-OHdG , 8-hydroxy-2’-deoxyguanosine; ORP, oxidation-reduction potential; TMC, total motile sperm count.

† Wilcoxon signed-rank sum test.

‡Wilcoxon rank-sum test

§ Median values (25th–75th percentile)
